# Integrating GWAS and Random Forest to Develop a Robust 5-SNP Panel for Genetic Sex Identification in Pacific White Shrimp (*Penaeus vannamei*)

**DOI:** 10.3390/ijms27219409

**Published:** 2026-10-22

**Authors:** Junyu Liu, Yan Xia, Pengcheng Li, Mianyu Liu, Xianhong Meng, Kun Luo, Juan Sui, Ping Dai, Qiang Fu, Jian Tan, Jiawang Cao, Baolong Chen, Xupeng Li, Jie Kong, Sheng Luan

**Affiliations:** State Key Laboratory of Mariculture Biobreeding and Sustainable Goods, Yellow Sea Fisheries Research Institute, Chinese Academy of Fishery Sciences, Qingdao 266071, China; liujy@ysfri.ac.cn (J.L.);

**Keywords:** *Penaeus vannamei*, sex identification, genome-wide association study, Random Forest, SNP panel

## Abstract

The Pacific white shrimp (*Penaeus vannamei*) exhibits pronounced sexual dimorphism in growth, making early and accurate sex identification crucial for targeted monitoring of sexual development and in-depth research into monosex aquaculture. However, morphological sex identification is unreliable at early developmental stages, and single-marker molecular assays are prone to diagnostic failures caused by genotyping errors or missing data. In this study, we developed a multi-SNP framework for sex prediction by integrating genome-wide association studies (GWAS) with Random Forest (RF) modeling. Analyzing 1244 individuals with a 55K SNP array, we identified 18 genome-wide significant SNPs and 10 candidate genes, including *SRRM1* and *SPSB3*, that may be associated with sexual differentiation. Subsequent multi-marker comparative analyses demonstrated that although the top-performing single marker yielded high accuracy, simulated genotyping errors and missing data caused significant drops in accuracy and elevated no-call rates, whereas multi-SNP panels exhibited substantially improved robustness. A stable performance plateau was reached when expanding the panel to 5 SNPs. The model achieved 100% classification accuracy in the two mixed-sex independent populations, ZJ and HN, and correctly identified all females in the all-female MK cohort. Overall, this study provides a robust multi-SNP tool for sex identification in *P. vannamei* and highlights the practical utility of machine learning in aquaculture.

## 1. Introduction

The Pacific white shrimp, *Penaeus vannamei*, commonly known as the South American white shrimp, is characterized by rapid growth, strong resistance to stress, wide salinity adaptability, and high farming yield. The total production of *P. vannamei* in 2024 was approximately 7.7 million tons, making it the highest in both value and production among single aquaculture species globally [1]. Alongside progress in larval rearing, nutrition, immunity, and genomic selection breeding programs [2,3,4,5], there has been a growing interest in sex determination research in aquatic animals [6]. As in other aquaculture species, the Pacific white shrimp exhibits sexual dimorphism, in which females may have a higher growth rate than males [7]. In *P. vannamei*, sexual dimorphism in growth has been reported to emerge as early as 50 days post-hatching and persist through the harvest stage, with a maximum weight difference of up to 20 g between females and males [8]. Therefore, conducting targeted tracking of sexual development during the early developmental stages of *P. vannamei* to elucidate the mechanisms underlying sex determination and to develop effective strategies for monosex culture is of considerable scientific and practical importance [6,9]. However, during the post-larval and early juvenile stages, shrimp lack distinct external morphological characteristics, making accurate sex identification impossible. The conventional manual method for sexing mature shrimp is labor-intensive and time-consuming, and for juvenile shrimp weighing less than 5 g (approximately 50 days of culture), the misidentification rate is extremely high [10]. In specific scientific contexts, such as full-life cycle tracking experiments and the exploratory investigations of sex reversal, genomic sex markers provide a valuable diagnostic tool.

Molecular marker-based sex identification represents a promising and efficient approach to overcome these limitations. To date, using high-density linkage mapping or genome-wide association studies (GWAS), multiple genes and genomic regions associated with sex determination have been identified in *P. vannamei* [7,11]. A high-density linkage map has been constructed for *P. vannamei*, in which a large region from 0 to 21.205 cM on linkage group (LG) 18 was significantly associated with sex [12]. Garcia et al. [7] reported that a sex-determining region was found in LG31 with 21 statistically significant SNPs. The most important SNP was previously identified as a sex-linked marker and was able to identify 99% of the males and 88% of the females. While previous studies have successfully developed single sex-linked molecular markers (e.g., Marker19299 or SNP_25749_180) and achieved sex identification via Sanger sequencing or HRM [13,14], these traditional approaches have notable limitations in large-scale practical breeding. Primarily, relying on a single or a few loci is highly susceptible to ‘single-point failures’—where amplification dropout or sequencing errors at a specific locus inevitably lead to irreversible misclassification. Furthermore, conventional PCR-based assays are labor-intensive, difficult to automate, and inefficient for high-throughput screening. Therefore, there is an urgent need to develop a rapid, accurate and robust method for sex identification in *P. vannamei*.

In recent years, Machine Learning (ML) approaches have gained immense popularity across disciplines, primarily due to their exceptional stability and robustness in processing complex biological data. Unlike traditional methods relying on single variables, ML algorithms, particularly ensemble methods such as Random Forest (RF) and XGBoost, leverage the joint analysis of multiple features to capture complex correlations and information redundancy. This mechanism endows the models with remarkable fault tolerance, ensuring highly stable and accurate predictions even in the presence of data noise, such as partial data missingness or genotyping errors. Benefiting from this strong resilience against interference, ML has been increasingly adopted in agriculture and aquaculture for applications including species identification, individual counting, morphometric measurement, and breeding management [15]. In the context of aquaculture sex identification, ML-assisted approaches have already demonstrated their potential to overcome the instability of single markers. For instance, using 15 sex-related SNPs, an XGBoost approach was employed for sex identification in rainbow trout, yielding a prediction accuracy of 98% [16]. However, a highly accurate and stable machine learning-derived multi-SNP panel, paired with a rapid detection method, has not yet been established for the large-scale sex identification of *P. vannamei*. RF is adept at handling discrete SNP predictors, accommodating potential non-linear interactions, and providing intuitive variable-importance metrics to prioritize candidate loci during panel construction, making it an ideal model for this application.

Therefore, the present study aims to develop a reliable multi-SNP panel and method for the genetic sex identification of *P. vannamei*. First, a GWAS was conducted to identify candidate sex-linked SNPs based on 1244 individuals. Subsequently, an RF algorithm was employed to select the most discriminative loci and construct a robust SNP panel. Based on the genotyping profiles of this panel, an RF-based predictive model was established and systematically validated in 3 populations with distinct breeding backgrounds. The establishment of this rapid and high-precision identification framework provides a powerful tool for early-stage sex tracking in juvenile shrimp, which may facilitate exploratory research on monosex culture in *P. vannamei*.

## 2. Results

### 2.1. Significant SNPs Associated with Sex Determination

**Figure 1:**
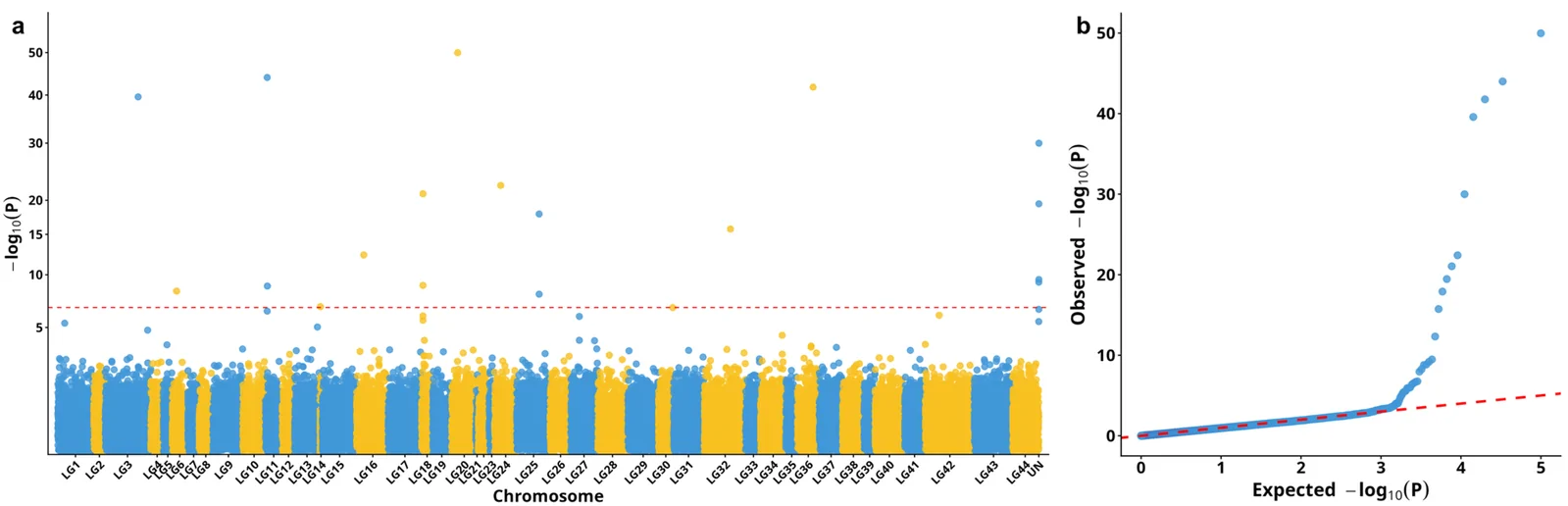
Results of the sex-related association analysis in *P. vannamei*. (**a**) Manhattan plots; (**b**) QQ plots. The dashed line represents the genome-wide significance threshold (*p* < 2 × 10^−7^).

To identify the SNPs associated with sex determination in *P. vannamei*, a genome-wide association study (GWAS) was conducted using the FarmCPU model (Figure 1). The genomic inflation factor (λ_GC_) was 0.93, indicating an acceptable control of confounding effects without notable signal inflation. Based on the predefined genome-wide significance threshold (*p* < 2 × 10^−7^), a total of 18 SNPs were identified as significantly associated with sex determination. As visualized in the Manhattan plot, these SNPs were in multiple linkage groups. Genomic annotation of the regions flanking the peak SNPs revealed 10 candidate genes, notably *SRRM1* and *SPSB3*, which are likely to play important roles in sex differentiation (Table 1).

### 2.2. SNP Panel of Sex Identification

**Figure 2:**
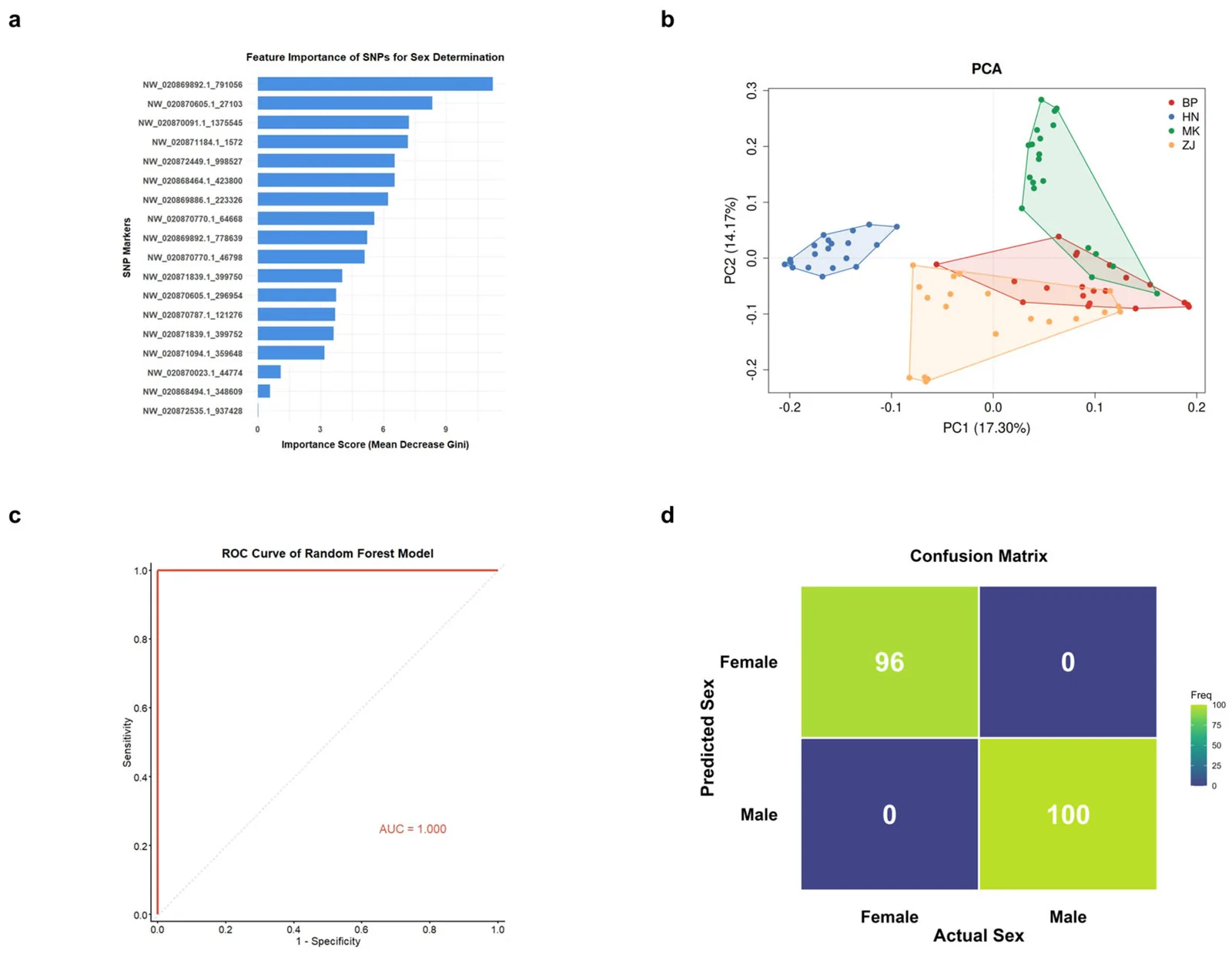
(**a**) The feature importance of significant SNPs using the Mean Decrease Gini index. (**b**) Principal component analysis (PCA) plot of the four populations, a random subset of 20 individuals per population is shown for clarity. (**c**) Receiver operating characteristic (ROC) curve with the corresponding area under the curve (AUC). (**d**) Confusion matrix for the ZJ population.

To translate the genome-wide signals into a practical and effective diagnostic tool, we employed a Random Forest (RF) algorithm for feature selection. The feature importance was quantified using the Mean Decrease Gini index, which measures the contribution of each SNP to the homogeneity of the nodes and leaves in the resulting RF model. As illustrated in the feature importance plot (Figure 2a), the predictive capacity varied among the markers. The SNP NW_020869892.1_791056 exhibited the highest discriminatory power, suggesting that it may play an important role in sex discrimination. To reduce model complexity and minimize potential noise from weakly associated markers, we initially considered SNPs with a Mean Decrease Gini score of ≥5.0 as candidate core highly informative markers. Based on this criterion, a subset of 10 primary SNPs (Table 2) was successfully screened out, which were subsequently utilized to construct the final optimized Random Forest model for sex prediction.

### 2.3. The Robustness of Single-Marker and Multi-Marker Panels

**Figure 3:**
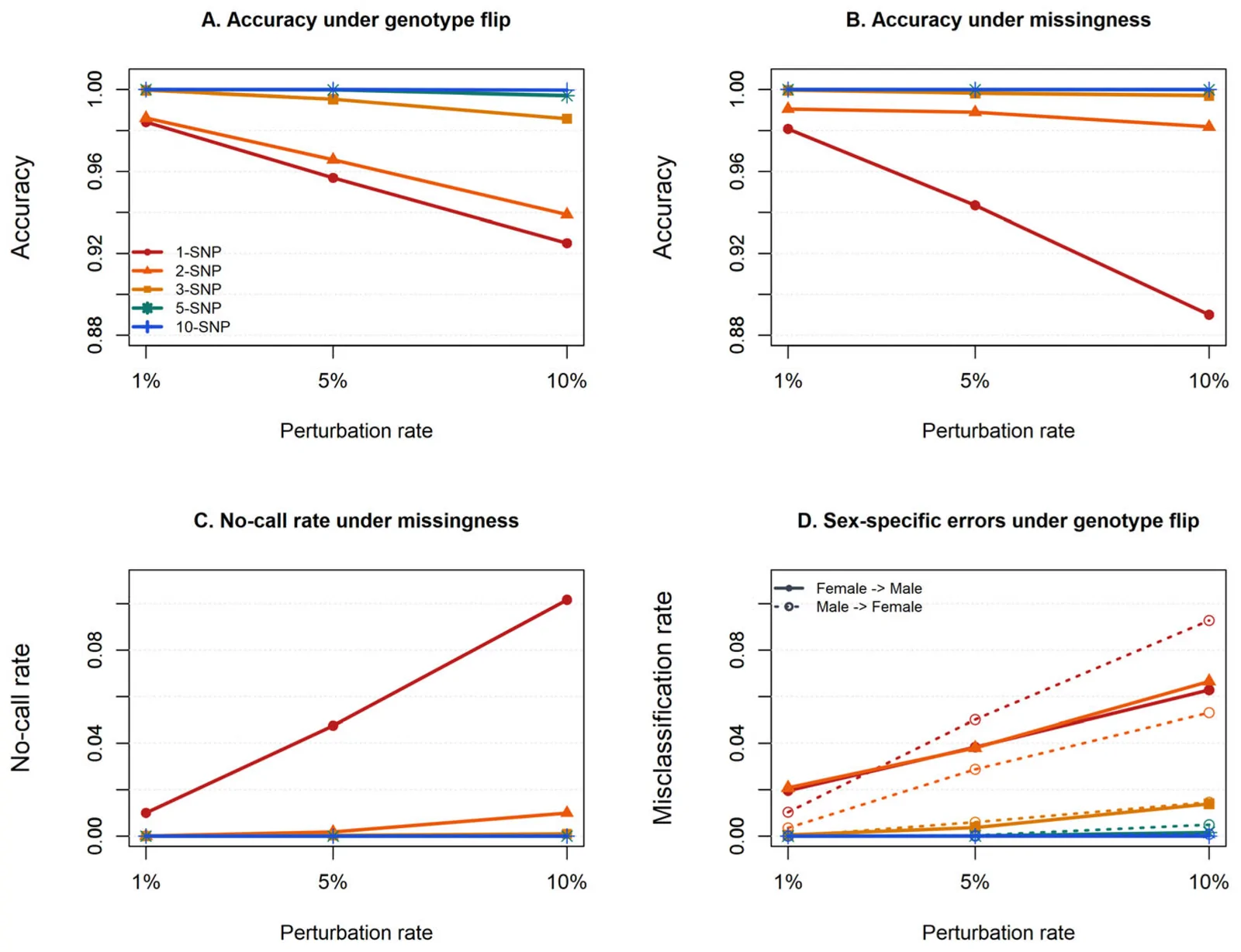
Robustness of single-marker and multi-marker sex-prediction panels under simulated genotype perturbation. (**A**) Accuracy under genotype flip. (**B**) Accuracy under genotype missingness. (**C**) No-call rate under genotype missingness, where no-call denotes samples that could not be assigned to either sex because the available marker information was insufficient for a confident classification. (**D**) Sex-specific misclassification rates under genotype flip, including female-to-male and male-to-female errors.

The results clearly demonstrate the distinct advantages of multi-marker panels in resisting noise (Figure 3): Under 5% genotyping errors: the diagnostic accuracies of the single-marker (1-SNP) and two-marker (2-SNP) models dropped to 0.9570 and 0.9653, respectively, whereas the 5-SNP and 10-SNP panels maintained high accuracies of 0.9996 and 1.0000. Under 10% genotyping errors: The single-marker accuracy plummeted to 0.9218 (0.9380 for 2-SNP), while the 5-SNP and 10-SNP models remained virtually unaffected (0.9971 and 0.9999, respectively). Under 10% data missingness: Data missingness substantially undermined the decision-making capacity of single-marker rules, causing the no-call rate to increase to 10.10% for 1-SNP and 1.03% for 2-SNP. In sharp contrast, both the 5-SNP and 10-SNP panels maintained a no-call rate of 0 and preserved an accuracy of 1. A stable performance plateau was reached when expanding the panel to 5 SNPs. Therefore, the 5-SNP Random Forest model was used for subsequent validation analyses.

### 2.4. Genotype Architecture of 5 Core SNPs in the Training Population

**Figure 4:**
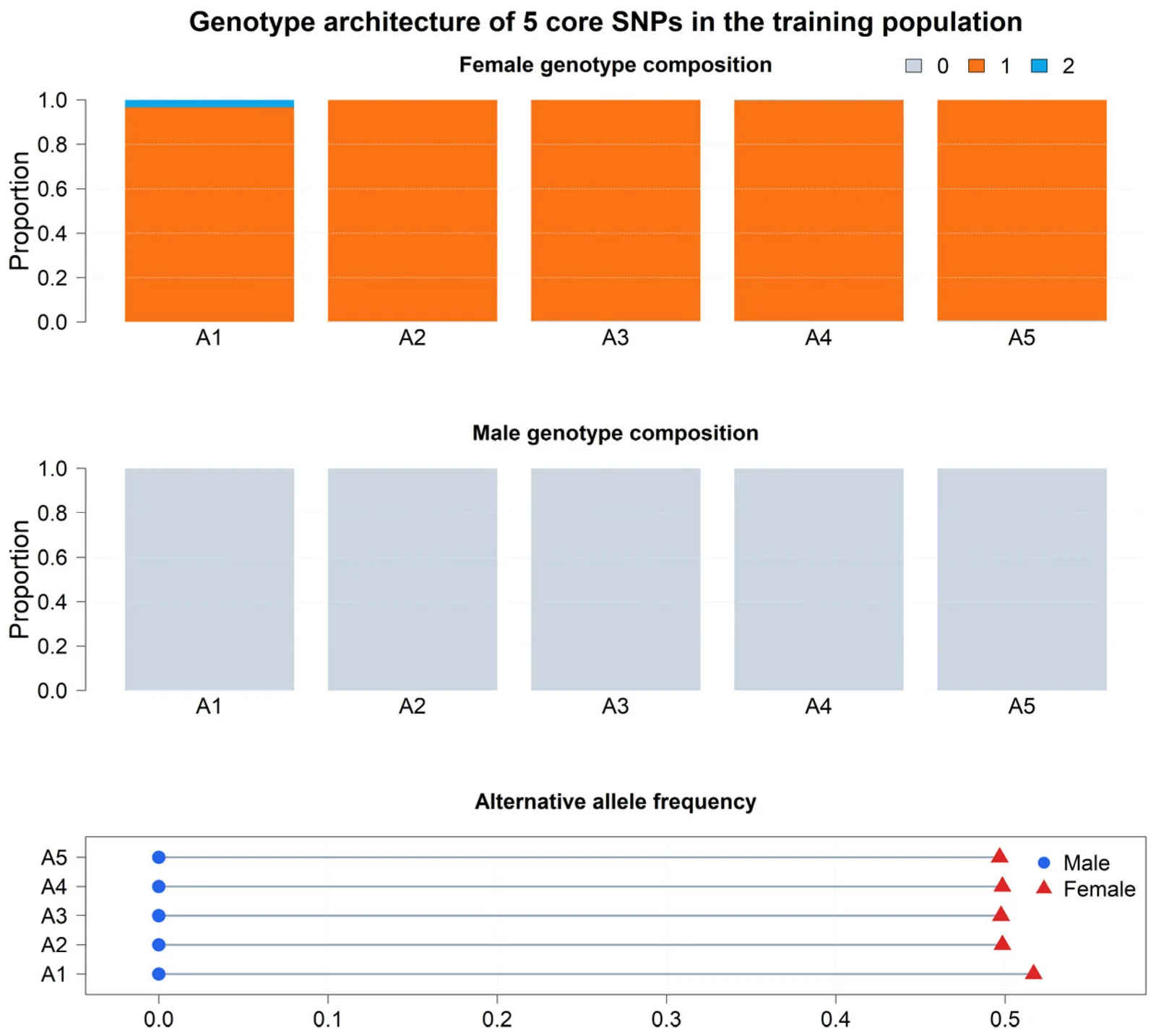
Genotype architecture of 5 core SNPs in the training population (BP) in *P. vannamei*.

The genotype composition, distribution counts, pairwise r^2^ values and alternative allele frequencies (AAFs) for each core SNP across males and females in the training population are shown in Figure 4, Figure S2 and Table S2. A highly distinct, sex-specific segregation pattern was observed across all 5 loci. In males, the genotype composition was virtually uniform, with most individuals displaying the reference homozygous genotype (encoded as ‘0′) across all 5 SNPs, and the AAF at these loci was near zero. Females exhibited a predominantly heterozygous genotype (encoded as ‘1′) for all 5 SNPs, with AAF values tightly clustered around 0.5. This segregation pattern, where males are homogametic and females are heterogametic, not only explains the high discriminative power of the Random Forest model but is also consistent with the hypothesis of a ZW/ZZ sex-determination system in *P. vannamei*. Furthermore, pairwise LD (*r*^2^) analysis across the core loci revealed extremely high correlation, with values ranging from 0.931 to 0.990 (Supplementary Figure S2).

### 2.5. Cross-Population Validation

Principal component analysis (PCA) revealed a clear genetic differentiation among the 4 populations, with each population forming a distinct cluster (Figure 2b). External validations across three populations of the ZJ, HN, and MK were performed to evaluate the generalized predictive capability and applicability of 5-SNP panel Random Forest model. The results showed that the diagnostic model achieved 100% classification accuracy in the two mixed-sex populations (ZJ and HN) and 100% sensitivity in identifying females in the all-female cohort (MK). Taking the ZJ population as an example, the ROC curve analysis yielded an outstanding Area Under the Curve (AUC) of 1.000 (Figure 2c), and the corresponding confusion matrix was consistent with this result, showing that all individuals were accurately classified with no false positives or false negatives (Figure 2d).

## 3. Discussion

In modern aquaculture, sex-specific molecular markers play a valuable role, particularly in sexually dimorphic species such as *P. vannamei*. Accurate sex determination at early developmental stages using molecular tools enables targeted monitoring of sexual development and exploratory physiological experiments. Furthermore, although the complete breeding pipeline for all-female stocks (such as generating functional neomales and putative WW superfemales) has not yet been established in *P. vannamei*, experimental investigations into physiological sex reversal, such as male-to-female reversal into neo-females via 17β-estradiol, have emerged as an active research hotspot to elucidate the underlying molecular mechanisms [17,18], thereby providing a theoretical foundation for future mono-sex breeding programs. Because sex-reversed individuals (e.g., neo-females with a ZZ genotype) are morphologically indistinguishable from normal genetic females (ZW), the sex-linked molecular markers developed in this study serve as an effective and indispensable diagnostic tool for their accurate identification.

Previous studies have made considerable progress in mapping the genetic loci for sex determination in *P. vannamei*. For instance, Yu et al. [12] pinpointed a genomic interval (0–21.205 cM) on linkage group 18 (LG18), highlighting three perfectly associated markers as promising candidates for sex differentiation. Subsequently, an independent GWAS encompassing 1952 shrimp by Jones et al. [11] revealed a cluster of 11 significant SNPs in strong linkage disequilibrium located on LG42.44 (0–12.7 cM). More recently, Garcia et al. [7] characterized a novel sex-associated region on LG31 harboring 21 significant SNPs. Notably, the top-performing variant in their study—a previously documented sex-linked marker—demonstrated a robust diagnostic accuracy of 99% for males and 88% for females. In this study, we performed a genome-wide association study utilizing 55K array data from 1244 *P. vannamei* individuals. In total, 18 SNPs and 10 candidate genes were identified. Compared to previous studies, the relatively lower marker density of the 55K SNP array utilized in our analysis likely limited the resolution to detect sharp association peaks in the Manhattan plot. Nonetheless, our study recovered sex-associated candidate genes congruent with earlier reports, supporting the reliability of our findings. For instance, consistent with the findings of Garcia et al., the serine/arginine repetitive matrix protein (SRRM1) gene was also identified in our analysis. In *Penaeus monodon*, transcriptomic analyses have revealed that this gene is downregulated in both testis and ovary tissues [19], while broader serine–arginine proteins are differentially expressed during testis maturation [20]. Furthermore, our analysis identified a significant signal (64668) on linkage group 18 (LG18), which resides very close to Marker64579, a sex-associated locus previously reported by Yu et al. The physical proximity of these independently identified markers lends further support to the involvement of this genomic region on LG18 in sex determination.

Importantly, our study identified a new candidate gene SPRY domain-containing SOCS box protein 3 (*SPSB3*). This identification was driven by a significant locus (791056) on linkage group 25 (LG25), which is located directly within the genomic region of this gene. Consisting of *SPSB1* through *SPSB4*, the *SPSB* (SPRY domain-containing SOCS box) family is broadly implicated in regulating immunity, stress tolerance, apoptosis, and growth in higher animals [21,22]. Gustavus, acting as the arthropod counterpart to vertebrate SPSB proteins, is extensively documented as a key driver of reproduction. In Drosophila melanogaster, Gustavus not only promotes fertility [23] but also critically controls germ cell differentiation by interacting with Vasa to ensure its proper embryonic accumulation [24]. Similar reproductive functions have been characterized in decapod crustaceans. Alternative splicing of Gustavus in *Neocaridina* denticulata sinensis generates two isoforms (NdGusX1 and NdGusX2) that are integral to vitellogenesis and the regulation of mature oocyte dimensions [25]. Additionally, extensive spatiotemporal expression analyses in *Macrobrachium nipponense* have linked Gustavus tightly to oocyte maturation and early embryonic development, validating its versatile regulatory capabilities [26]. Collectively, the identification of these candidate genes provides a valuable theoretical basis and molecular targets for sex-controlled breeding in *P. vannamei* and other crustacean species.

In this study, phenotypic sex was analyzed as a binary trait using genome-wide association study with the FarmCPU algorithm [27]. By iteratively implementing fixed and random effect models with pseudo-QTNs incorporated as covariates, FarmCPU decouples testing markers from kinship, thereby effectively eliminating false positives arising from cryptic relatedness. Applying linear mixed model (LMM)-based frameworks (including FarmCPU) to binary traits—such as sex determination, disease challenge survival/mortality, and shell/body coloration—has been widely utilized in aquatic genetics (e.g., in fish [28], crustaceans [29], and mollusks [30]), successfully uncovering a series of critical candidate genes for economically important traits. In comparison, logistic regression directly models the liability distribution of binary outcomes via non-linear link functions and naturally estimates odds ratios (ORs), making it theoretically more congruent with discrete phenotypes. Although FarmCPU inherently bears theoretical limitations due to its underlying linear model framework, its application here is methodologically sound given our balanced female-to-male ratio (~1:1, where linear approximations retain high statistical power). Moreover, subsequent validation experiments confirmed the diagnostic accuracy of the identified loci, corroborating the high biological reliability of the candidates screened by this model. Future studies could further integrate specialized logistic mixed models to refine candidate variant discovery for discrete traits.

Beyond identifying sex-associated genomic regions, translating these GWAS findings into practical breeding tools is a pivotal goal. In this study, we developed a sex-identification panel and RF method based on 5 highly significant SNP markers. Pairwise linkage disequilibrium (LD) analysis revealed exceptionally high correlation among the five selected SNPs (r^2^ = 0.93–0.99), despite their provisional assignment to different linkage groups or unassigned scaffolds in the reference assembly. This near-complete co-segregation indicates that rather than representing multiple independent sex-determining loci, these five SNPs are highly likely to collaboratively track the same underlying sex-linked genomic signal. Regarding this discrepancy between physical assignment and strong genetic linkage, we consider that several technical and biological factors may be involved. Foremost among these are the assembly quality and scaffolding limitations of the current reference genome. The currently available *P. vannamei* reference genome (ASM378908v1) was primarily assembled from short-read sequencing data. Like many decapod genomes, the *P. vannamei* genome exhibits exceptionally high heterozygosity and an extensive proportion of repetitive elements (>50%). To date, a telomere-to-telomere (T2T) or gap-free chromosome-level assembly remains unavailable for this species. During the draft construction and pseudo-chromosome anchoring phases, ambiguities and orientation errors in scaffold placement onto linkage groups were likely inevitable. Consequently, genomic segments or scaffolds that genuinely belong to a single sex-determining chromosome or region could have been erroneously misassigned to distinct autosomal linkage groups. Additionally, biological dynamics characteristic of sex-determining regions—such as sex-linked sequence duplications, paralogous expansions, and recombination suppression—as well as potential probe cross-hybridization, could cause sequences originating from the same sex-linked genomic block to align artificially across disparate scaffolds. Genetically, these five SNPs act as a unified, tightly linked haplotype block, which provides a coherent biological rationale for their uniformly high diagnostic accuracy in subsequent validation assays.

The use of genomic SNP profiles for sex prediction has been increasingly explored in aquatic species. Earlier studies have explored iterative Bayesian approaches for this purpose. In such models, an initial prior probability is typically set at 0.5 for the first iteration and is subsequently updated using the posterior probabilities derived from preceding rounds. Utilizing this framework, Toli et al. [31] successfully achieved high prediction accuracy when validating with 286 phenotypically sexed sticklebacks. However, this methodology is exquisitely sensitive to both the quality and the input sequence of the markers during the iterative solving process. Consequently, if uninformative or noisy SNPs are introduced during the initial rounds, the resulting posterior distribution can become heavily skewed, leading to substantial biases or misclassifications in the final sex prediction. To overcome the limitations of traditional models, machine learning algorithms have been recently introduced. For instance, in rainbow trout, Kudinov et al. [16] used the Extreme Gradient Boosting (XGB) approach to predict sex from genomic data, achieving a high accuracy of 98%. Notably, the efficacy of XGB relies heavily on the genetic similarity between the training and testing cohorts. Applying the model to genetically distant populations significantly impair accuracy, as evidenced by a 21% misclassification rate observed in cross-dataset predictions. As an ensemble learning method based on bootstrap aggregating (bagging), RF constructs hundreds of independent decision trees in parallel. It achieves robust performance through its parallel voting mechanism. In our study, the RF-selected SNP panel retained an accuracy above 99.9% even under simulated 10% missingness or 10% genotyping error. In this study, the observed enhancement in fault tolerance is primarily attributable to marker redundancy, while the primary utility of Random Forest lies in its capacity to conveniently integrate these redundant marker inputs without rigid parametric assumptions, thereby providing a standardized and reproducible decision framework for high-throughput automated diagnostic workflows.

Several non-destructive and non-invasive sex identification methodologies for aquatic species have been proposed in recent years. These innovative approaches typically integrate morphological dimorphism, image recognition, and deep learning frameworks to predict sex based on external phenotypic traits. For instance, convolutional neural networks have achieved accurate sex classification in zebrafish based on body images [32], while assisted ultrasound imaging has been applied to automate sex sorting in commercial tilapia breeding operations [33]. Gao et al. [34] proposed a combined DL and RF framework for the non-invasive early sex identification of hybrid sturgeons. By utilizing DL to pinpoint morphologically distinct regions and RF to screen for key sex-specific features, their multi-view fusion DL model reached an optimal accuracy of 89.0%. While these advanced deep learning and computer vision technologies provide an innovative, cost-effective, and efficient avenue for sex identification in aquatic animals, their applicability is inherently limited during early life stages prior to morphological sex differentiation. Looking forward, propelled by the exponential explosion of multi-omics data and the rapid advancement of big data analytics, we are ushering in a new era of artificial intelligence (AI)-driven breeding. At present, ML/DL frameworks are increasingly being embedded into comprehensive genomic selection (GS) pipelines, facilitating critical tasks such as precise phenotype measurement [35], genotype imputation [36], GS prediction [37], and the deployment of intelligent breeding agents [38]. Although these technological integrations still face numerous practical challenges, they represent the future of the industry. Ultimately, as these technologies continue to mature, they hold great promise for accelerating the genetic improvement and sustainable development of aquaculture species.

## 4. Materials and Methods

### 4.1. Data Collection

A total of 1244 individuals, originating from a breeding population from BLUP Aqua breed Co., Ltd. in Weifang, Shandong Province, China, were selected to construct the training population (BP) for the model. All selected individuals had a body weight of greater than 7 g to ensure accurate morphological identification. Phenotypic sex was determined via manual visual inspection of the external genitalia, yielding a balanced dataset consisting of 621 males and 623 females. For subsequent machine learning modeling, the phenotypic sex of everyone was computationally encoded as a binary dependent variable, where males were assigned a value of 1 and females were assigned a value of 0.

### 4.2. Genotyping

Genotypic data were obtained for all individuals in the training population using the 55K SNP panel of “Yellow Sea Array No.1” [39]. SNPs with a minor allele frequency (MAF) <0.05 and call rate <0.90 were removed, and individuals with call rates <0.80 were excluded. All missing genotypes were imputed using BEAGLE v4.0 with default parameter settings [40]. Finally, 49,886 SNP markers and 1244 individuals remained for further analysis. To transform the genomic data into a mathematically processable format for machine learning algorithms, the genotypes were converted following an additive genetic model. Specifically, genotypes were numerically encoded based on their alignment with the *P. vannamei* reference genome (ASM378908v1): homozygous genotypes identical to the reference allele were encoded as ‘0′, heterozygous genotypes as ‘1′, and homozygous variant (alternative) genotypes as ‘2′.

### 4.3. Genome-Wide Association Study (GWAS) and Candidate QTLs

Genome-wide association analyses were executed utilizing the FarmCPU (Fixed and random model Circulating Probability Unification) algorithm implemented in the rMVP software 1.0.4 [27,41]. To effectively mitigate confounding biases arising from population stratification and genetic relatedness, the top three principal components (PCs) and a kinship matrix were incorporated into the model as fixed and random covariates (Figure S1; Kinship Matrix Table S1), respectively [42]. The significance thresholds were established based on a stringent Bonferroni correction (α = 0.01). The genome-wide significance threshold was set at *P* = 2 × 10^−7^ (−log10(*p* value) = 6.69, calculated as 0.01/49,886, where 49,886 represents the total number of quality-filtered SNPs) [43]. Visualization of the GWAS results, including the generation of Manhattan and quantile–quantile (QQ) plots, was performed using the R package CMplot 4.5.1 [41]. For downstream functional annotation, genomic regions spanning 50 kb upstream and downstream of the identified significant SNPs were systematically scanned to screen for potential candidate genes involved in sex determination.

### 4.4. Construction of the Random Forest (RF) Classification Model

A RF classification model was implemented in R using the randomForest package based on the encoded genotype and phenotype datasets. The RF model was constructed based on the bootstrap aggregating (bagging) technique. The 18 genome-wide significant SNPs identified from the GWAS were utilized as the input feature set for the Random Forest (RF) model. Specifically, the training population was repeatedly sampled with replacement (bootstrapping) to generate multiple training subsets. A total of 200 independent decision trees (ntree = 200) were grown to form the random forest ensemble. During the construction of each decision tree, at every node split, a random subset of 3 SNP markers (mtry = 3) was evaluated. The optimal marker and its corresponding genotypic threshold were selected based on the minimization of the Gini index (Gini impurity). The node splitting process continued iteratively until all samples within a terminal node belonged to a single sex class (pure node) or could not be further divided. Ultimately, the ensemble of 200 trees established complex mapping relationships between specific genotypic configurations (0, 1, 2) and sex (0, 1) to generate the final prediction. To evaluate the association strength between specific SNP markers and sex determination, the relative feature importance (weight) of each SNP was calculated based on its contribution to reducing Gini impurity across the forest.

### 4.5. Evaluation Robustness of Single-Marker and Multi-Marker Panels

To evaluate whether expanding marker numbers provides superior robustness over simple single-marker diagnostics, we benchmarked nested SNP subsets on BP population. The 10 core SNPs were first ranked by their individual classification accuracy in the BP population, defining four nested panels: 1-SNP, 2-SNP, 3-SNP,5-SNP, and 10-SNP. Baseline performance was evaluated in the absence of noise. Subsequently, two independent perturbation scenarios were introduced across the genotype matrix: genotype flipping and genotype missingness, each tested at probabilities of 1%, 5%, and 10% per genotype call. For a given panel containing k SNPs in a dataset of n individuals, each simulation comprised n × k genotype calls, and each genotype call was independently perturbed with probability p, yielding an expected p × n × k perturbed calls per simulation. Under the missingness scenario, selected genotype calls were set to missing. Under the genotype-flip scenario, each selected non-missing genotype call was randomly replaced by one of the other two possible genotype states. Each perturbation condition was repeated for 200 independent iterations, and overall accuracy, no-call rate, and sex-specific misclassification rates were recorded. A sample was considered uncallable only when all SNPs in the current panel were missing; overall accuracy was calculated across all individuals, with uncallable samples counted as not correctly classified.

### 4.6. Validation in Independent Population

To evaluate the cross-population robustness of the identified core SNPs for sex determination, the Random Forest optimal model trained on the BP was directly applied to three external validation populations with distinct breeding backgrounds for testing. These three independent validation cohorts encompass distinct geographic origins and breeding backgrounds: (1) the ZJ population from Wenzhou, Zhejiang Province, consisting of 196 individuals (96 females and 100 males); (2) the HN population from Dongfang, Hainan Province, comprising 124 individuals (63 females and 61 males); and (3) the MK population from Qingdao, Shandong Province, which served as a single-sex test group containing 63 individuals (all females). MK is a commercial breeding population selected for high disease resistance, with directional selection focused on pathogen resistance and survival rate. ZJ and HN are fast-growth selection populations. We performed genetic structure analysis of four populations using PLINK v1.9 --pca.

## 5. Conclusions

In conclusion, this study successfully identified the sex-associated SNPs and candidate genes in *P. vannamei*. By synergizing these findings with a Random Forest machine learning algorithm, we developed a sex prediction model based on a 5-SNP panel. The results showed excellent performance in the external validation populations and simulation tests conducted in this study. This rapid and robust molecular tool effectively overcomes the limitations of early-stage morphological sexing and single-marker assays.

## Figures and Tables

**Table 1:** Details of candidate genes identified in GWAS.

Gene ID	Linkage Group	Scaffold	Location (bp)	Gene Name	Region	Gene Annotation
LOC113828906	LG 3	NW_020872449.1	982,862–994,750	*SMPD-like*	upstream	sphingomyelin phosphodiesterase
LOC113824360	LG 6	NW_020871094.1	385,030–411,925	*Toll*	downstream	protein toll-like
LOC113824354	LG 6	NW_020871094.1	366,220–377,722	*U2AF35*	downstream	U2 small nuclear ribonucleoprotein auxiliary factor 35 kDa subunit
LOC113820141	LG 11	NW_020870605.1	36,852–43,964	*COMMD7*	downstream	COMM domain-containing protein 7-like
LOC113820152	LG 11	NW_020870605.1	15,000–18,458	*Cutlet*	upstream	chromosome transmission fidelity protein 18 homolog
LOC113829448	LG 16	NW_020872535.1	973,398–978,651	*Nad kinase*	downstream	NAD kinase-like
LOC113821533	LG 18	NW_020870770.1	82,405–90,531	*PTPRF*	downstream	receptor-type tyrosine-protein phosphatase F
LOC113813571	LG 25	NW_020869892.1	704,517–749,454	*SRRM1*	upstream	serine/arginine repetitive matrix protein 2-like
LOC113813591	LG 25	NW_020869892.1	779,250–793,683	*SPSB3*	intragenic	SPRY domain-containing SOCS box protein 3
LOC113829473	LG 36	NW_020870091.1	1,399,457–1,399,951	*Dachs*	downstream	unconventional myosin-IXb-like dachs

**Table 2:** Genomic information and feature weights of the 10 core SNPs comprising the sex-determination panel.

ID	Linkage Group	Scaffold	Position	Ref/Alt	MAF	*p* Value	Weight	Accuracy
A1	LG25	NW_020869892.1	791,056	A/G	0.259	1.24 × 10^−18^	9.67	1
A2	LG11	NW_020870605.1	27,103	G/T	0.249	9.98 × 10^−45^	7.79	0.9984
A3	LG3	NW_020872449.1	998,527	C/T	0.249	2.60 × 10^−40^	7.68	0.9976
A4	LG36	NW_020870091.1	1,375,545	C/T	0.249	1.69 × 10^−42^	7.66	0.9976
A5	UN	NW_020871184.1	1572	G/C	0.249	1.01 × 10^−30^	7.09	0.9968
A6	LG20	NW_020868464.1	423,800	T/A	0.247	1.06 × 10^−50^	6.95	0.9944
A7	LG25	NW_020869892.1	778,639	G/A	0.246	1.09 × 10^−8^	6.00	0.9936
A8	LG18	NW_020870770.1	64,668	A/T	0.250	8.99 × 10^−22^	5.90	0.9936
A9	LG32	NW_020869886.1	223,326	G/A	0.249	1.81 × 10^−16^	5.71	0.9928
A10	LG18	NW_020870770.1	46,798	C/T	0.244	1.38 × 10^−9^	5.10	0.9912

## Data Availability

Necessary subsets of supporting data can be provided to researchers upon reasonable request for non-commercial validation and collaborative research.

## Supplementary Materials

The following supporting information can be downloaded at: https://www.mdpi.com/article/10.3390/ijms27219409/s1.
