# Supplementary material for: Integrating GWAS and Random Forest to Develop a Robust 5-SNP Panel for Genetic Sex Identification in Pacific White Shrimp (Penaeus vannamei)
Source: Int J Mol Sci. 2026 Oct 22;27(21):9409. doi: 10.3390/ijms27219409 (PMC13763122; doi:10.3390/ijms27219409)

Figure S1. The principal component analysis (PCA) of BP population

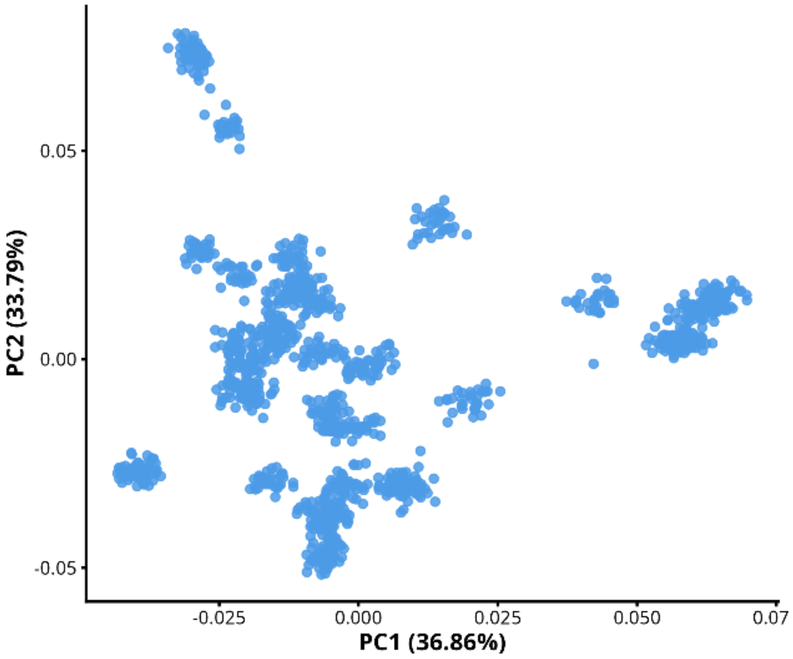

Figure S2. The pairwise LD (r²) values for the top 5-SNP panel

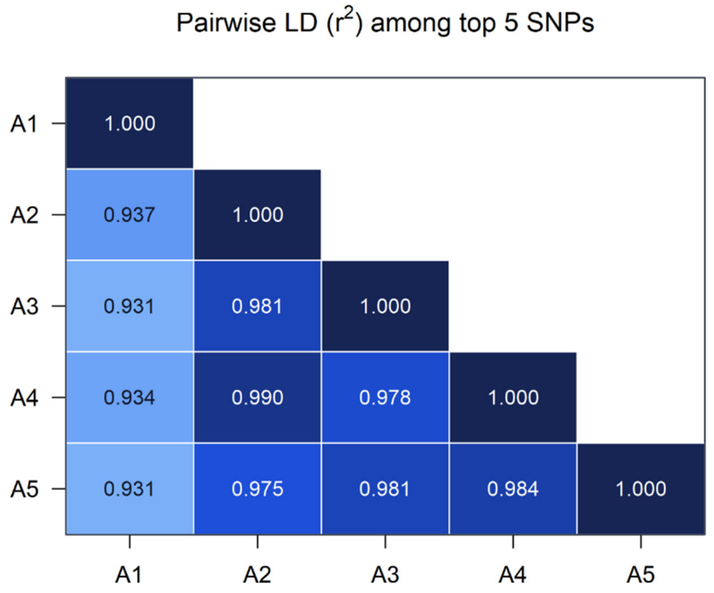

Supplement: Supplementary file 1 [file ijms-27-09409-s001.zip › Supplementary Figure.pdf]
