# Supplementary material for: Integrating GWAS and Random Forest to Develop a Robust 5-SNP Panel for Genetic Sex Identification in Pacific White Shrimp (Penaeus vannamei)
Source: Int J Mol Sci. 2026 Oct 22;27(21):9409. doi: 10.3390/ijms27219409 (PMC13763122; doi:10.3390/ijms27219409)
Supplement: Supplementary file 1 [file ijms-27-09409-s001.zip › Supplementary Table S2.pdf]

Table S2. The genotype distribution counts for each core SNP across male and female individuals.

SNP	Male 0/0	Male 0/1	Male 1/1	Female 0/0	Female 0/1	Female 1/1
A1	621	0	0	0	602	21
A2	621	0	0	2	621	0
A3	621	0	0	4	619	0
A4	621	0	0	3	620	0
A5	621	0	0	3	619	1
